# Nonagenarians qualify for total knee arthroplasty: a report on 329 patients from the Swedish Knee Arthroplasty Register 2000–2016

**DOI:** 10.1080/17453674.2018.1530173

**Published:** 2018-10-18

**Authors:** Erdem A Sezgin, Otto Robertsson, Annette W-Dahl, Lars Lidgren

**Affiliations:** a Gazi University, Faculty of Medicine, Department of Orthopedics and Traumatology, Ankara, Turkey;;; b Lund University, Faculty of Medicine, Department of Clinical Sciences Lund, Orthopedics, Lund, Sweden;;; c The Swedish Knee Arthroplasty Register, Lund, Sweden

## Abstract

Background and purpose — The nonagenarian (those aged 90 years and older) population is expected to double in the next 20 years. This demographic age quake may have a significant impact on the incidence of total knee arthroplasty (TKA), although current literature provides limited data. We examined death and revision rates, patient-reported outcomes (PROs) and bias on patient selection of nonagenarian patients operated on with TKA for osteoarthritis (OA) between 2000 and 2016.

Patients and methods — The Swedish national knee arthroplasty register was used to identify 329 nonagenarians (mean age, 92 years). Each patient was followed-up until death or the end of 2017. PRO data of 22 of these patients were compared with 65- to 74-year-old patients operated in 2015, from the same register.

Results — 5 patients (1.5%) died within 90 days and 23 (7%) patients died within 365 days after TKA. 8 patients (2.4%) developed knee complications that needed revision. For patients followed for 5 and 10 years, more than 50% and 10%, respectively, lived without being revised. The patients had statistically significant improvements in PROs, not significantly different from the younger SKAR cohort. However, the material is small and this statistical finding does not preclude that there may be clinically relevant differences. TKA incidence was different amongst the 21 counties in the country (range, 0–5.1/10,000).

Interpretation — Our study suggests that nonagenarians with knee OA qualify for TKA, having similar outcomes to younger patients. The data presented may help surgeons and patients assessing the risks and outcome associated with the procedure.

The nonagenarian population in Sweden was 95,300 (0.94%) in 2017 and is expected to rise to 199,900 (1.7%) in 2040 (Statistics Sweden). This demographic age quake has a substantial impact on the prevalence of symptomatic osteoarthritis (OA) (Nemes et al. 2015). Amongst OA of weight-bearing joints, knee OA has the most pronounced correlation with age. It affects one-fourth of the reported 56- to 84-year-old individuals in a population study from southern Sweden (Turkiewicz et al. 2014). The demand for primary total knee arthroplasty (TKA) is on the rise all round the world and this trend is expected to continue in the future (Koh et al. 2013, Culliford et al. 2015, Kurtz et al. 2014, Nemes et al. 2015, Inacio et al. 2017, Niemelainen et al. 2017). In studies reporting the outcome of the oldest knee arthroplasty patients, the cut-off has often been 85 years and older (Laskin 1999, Biau et al. 2006, Easterlin et al. 2013, Williams et al. 2013). With a focus on nonagenarians, we have found only 4 studies, all with low patient numbers (12–42) which include data from the 1970s until early 2000s (Belmar et al. 1999, Joshi and Gill 2002, Pagano et al. 2004, Karrupiah et al. 2008) (Table 1).

**Table 1.** Summary of demographics and outcome data of current study and relevant literature

Study characteristics	Present study	Karrupiah et al.	Pagano et al.	Joshi and Gill	Belmar et al.
Year of publication		2008	2004	2002	1999
Timespan	2000–2016	1990–2006	1970–1997	1976–1999	1983–1997
Country of data	Sweden	UK	USA	USA	USA
Demographics					
Number of patients (knees)	329 (359)	42 (42)	34 (44)	18 (20)	12 (15)
Age mean (range)	92 (90–101)	90 (90–91)	92 (90–102)	91(90–93)	92 (90–96)
Female sex (%)	69	N/A	68	72	75
Months of follow-up (range)	50 (0.2–145)	90	48	62 (1–152)	N/A (6–30)
Diagnosis of OA (%)	100	100	91	100	80
Outcomes					
Death rate 90 days, n (%)	5 (1.5)	0 (45 days)	1 (3)	1 (5.6)	0
Death rate 1st year, n (%)	23 (7)	6 (14.3)	N/A	2 (11.1)	N/A
Mean survival after TKA					
First revision, n (%)	8 (2.4)	N/A	0	1 (5.6)	0
Mean length of stay (days)	6.2	11	13.5	10.1	15
PROs	KOOS	KSS Pain	KSS Pain	KSS Pain	KSS Pain
	Pain 47–82	25–81	30–86	45–95	2–49
	ADL 51–76				
	QoL 28–75				
	EQ-VAS	WOMAC	KSS Function	KSS Function	KSS Function
	71–76	62–41	29–38	28–53	26–33
	VAS satisfaction		Satisfaction		
	21/22		33/34		

ADL = activities of daily life function, QoL = quality of life, N/A = not available.

In this study we evaluated the nonagenarians in Sweden operated on with TKA for OA between 2000 and 2016 on 5 aspects: (1) the demographics, (2) early/total death rates (3) revision rate and reasons for revision, (4) patient-reported outcome (PRO), (5) difference in TKA incidence among the counties in Sweden.

## Patients and methods

Patients aged 90 years and older who had undergone primary TKA for OA between January 1, 2000 to December 31, 2016 were identified in the Swedish Knee Arthroplasty Register (SKAR).

Initiated in 1975 by the Swedish Orthopedic Association, the SKAR is the world’s first national arthroplasty register. The SKAR has high completeness and correctness of data (SKAR 2017).

Patient demographics including age at surgery, date of surgery, sex, and county of residence were identified from the SKAR as well as data for outcome measures including current age or date of death, revisions, and reasons for these procedures. In the SKAR, revisions are defined as a new operation in a previously resurfaced knee in which 1 or more of the components are exchanged, removed, or added (including arthrodesis and amputation) (SKAR 2017). All the identified patients were followed-up until death, or until December 31, 2017. The proportion of death within 90 days and 1 year after the TKA was assessed. Data from the Swedish life tables was used to compare 1-year death rates and cumulative death rates of the TKA patients included with the national average of the same age group in 2016 (Human Mortality Database).

Revisions, reasons for, and time of revisions and consequent operations (if these occurred) were compiled and revision rate was calculated.

We identified PROs in patients 90 aged years and older operated on with TKA for OA from the SKAR PROM database in addition to the aforementioned data. We included patients operated between 2009 and 2015, but excluded the second knee if both knees had an arthroplasty within 1 year and the left knee if simultaneous surgery was performed (1 patient). The PROs of this sub-cohort of patients were then compared with those of the 65- to 74-year-old primary TKA patients operated on for OA during 2015 (SKAR 2017). The SKAR PROM project started in 2009 with patients undergoing knee arthroplasty in southern Sweden. Since then, hospitals from other regions have entered the PROM project. In 2015 approximately 28% of all TKAs for OA were included in the project that had both preoperative and 1-year postoperative PRO data and the response rate was 72% in 2015 for the 65- to 74-year-old cohort (SKAR 2017). As PROs, we used the Knee injury and Osteoarthritis Outcome Score (KOOS), the EuroQol-visual analogue scale (EQ-VAS) and the visual analogue scale for patient satisfaction (VAS–satisfaction) (Roos et al. 1998, EuroQol Group 2015). Considering the difference in outcome between the groups, ≥ 8 points in the KOOS and ≥15 mm in EQ-VAS was considered a clinically relevant difference for statistically significant results. In addition, the KOOS was converted to the Western Ontario and McMaster Universities Osteoarthritis Index (WOMAC) to be able to classify each patient as Osteoarthritis Research Society International– Outcome Measures in Rheumatology (OMERACT–OARSI) responder or not at 1 year based on a combination of absolute and relative changes in WOMAC pain, function, and total scores (Pham et al. 2004). The outcome at 1 year was dichotomized into ‘responders’ and ‘non-responders’ according to these criteria (Pham et al. 2004). Patient satisfaction with the arthroplasty surgery 1 year postoperatively was evaluated using a 0–100 scale (VAS) in which 0 indicates the highest imag¬inable satisfaction and 100 the worst imaginable satisfaction. The satisfaction (VAS) score was categorized into 5 groups: very satisfied (0–20), satisfied (21–40), moderately satisfied (41–60), unsatisfied (61–80), and very unsatisfied (81–100). In routine practice, PROs are collected 2 to 6 weeks prior to surgery at the outpatient visit. 1 year postoperatively, the same questionnaire is mailed to the patients together with the VAS–satisfaction questionnaire.

Data for the length of stay (LoS) were only accessible for the region of Skane. Considering that LoS changed over the years, the data from only the last 10 years (January 1, 2007–December 31, 2016) were used for analysis to better reflect modern trends. LoS data were then compared between nonagenarians and the rest of the TKA population from the same period.

To compare patient selection bias between counties in Sweden, we used population data and number of TKAs for OA in each county to calculate incidence ratios.

## Statistics

Life-table or actuarial analysis was used to access the cumulative death rate. Student’s 2-sample t-test was used to compare the mean age at surgery between sexes for nonagenarians. Welch’s t-test was used for comparisons of the KOOS and the EQ-VAS between the 2 age cohorts considering unequal variances and unequal sample sizes with assumption of normal distribution. The normality of the PRO data was controlled using the 2-sample Kolmogorov–Smirnov test for equality of distribution functions and the distribution was found to be normally distributed for all PRO variables except for the preoperative KOOS symptoms. For analysis of proportions of OMERACT–OARSI responder and satisfaction with the surgery, a chi-square test was used for comparisons. The cumulative revision rate was calculated using the STATA Life-table method using one-month intervals. Statistical analyses were carried out using STATA (Stata Statistical Software: Release 15; StataCorp LLC, College Station, TX, USA).

## Ethics, funding, and potential conflicts of interest

The data gathering from the Swedish Knee Arthroplasty Register was approved by the Ethics Board of Lund University (LU20-02). The authors received no specific funding for this work. No conflicts of interest were declared.

## Results

**Figure 1.**
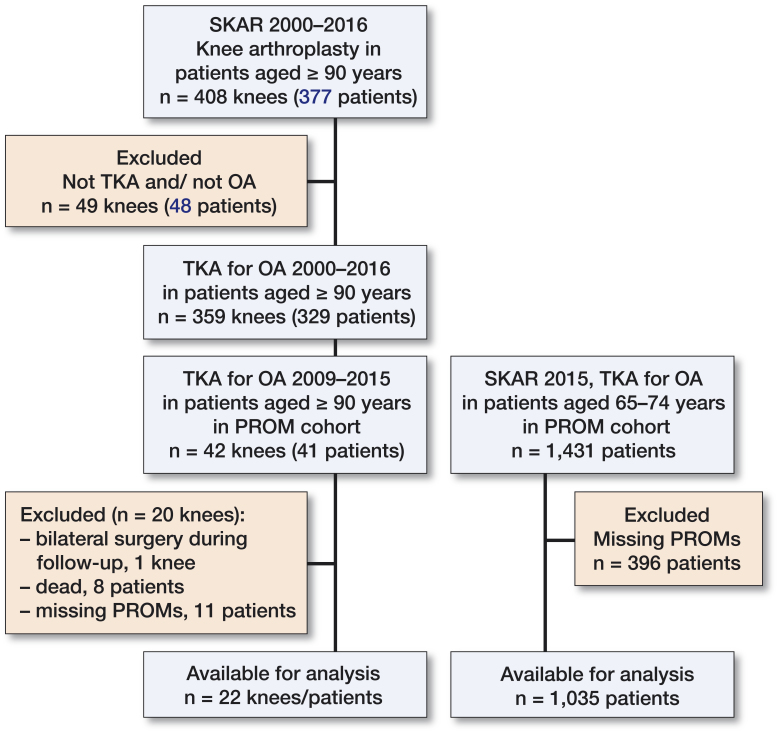
Flow diagram on the selection of patients.

Among 377 patients (408 knees) aged 90 years and older who had undergone knee arthroplasty, 329 patients with 359 primary TKAs inserted for OA were included in our study (Figure 1). 31 patients had bilateral TKAs. 227 (69%) patients were women; the mean age at surgery (first knee in the case of bilateral knees) was 92 years (90–101) and this was similar between women and men. The mean length of follow-up was 50 months (0.2–145).

**Figure 2.**
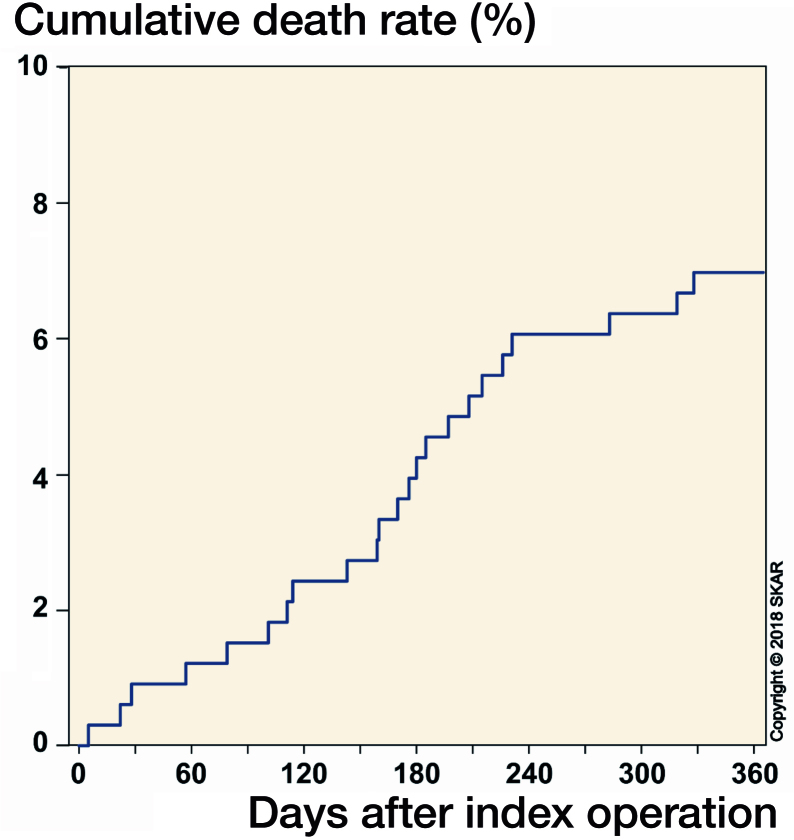
Death rate within 365 days after primary surgery.

5 patients (1.5%) died in the first 90 days postoperatively and 23 patients (7%) died within 365 days after the TKA surgery (Figure 2).

**Figure 3.**
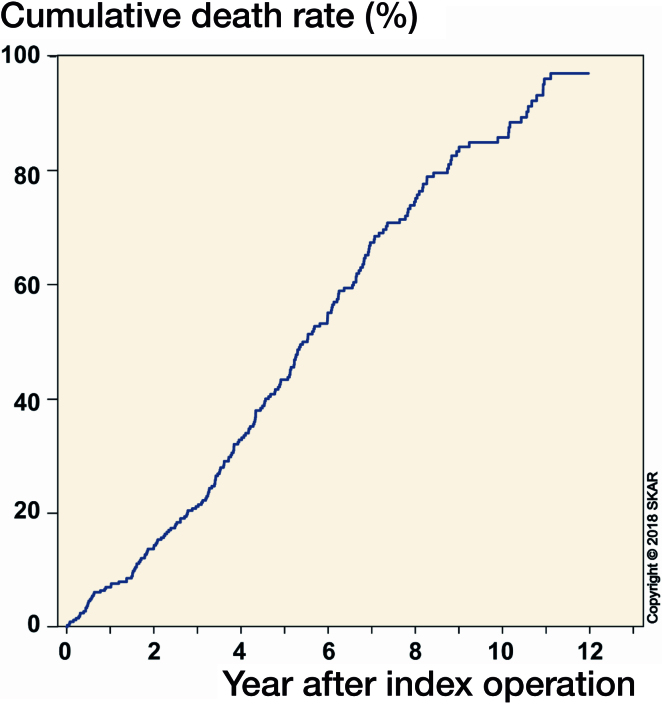
Cumulative death rate.

Up to December 31, 2017, 214 patients had died, leaving 115 alive. According to the cumulative death rate calculations, more than 50% of the patients had lived more than 5 years with their TKA and more than 10% had lived more than 10 years after the primary TKA (Figure 3).

2 patients who had bilateral TKAs had the implants inserted simultaneously. One was 92 years and lived for 1 year and 8 months. The other was 95 years and lived for 4 years and 11 months. The oldest patient operated was 101 years old when he received his second TKA (the first being replaced 12 years earlier). He lived for another 1 year and 4 months. None of the arthroplasties in these 3 patients were revised.

8 patients (2.4%) developed complications that needed revision. The revisions were mostly early (from 1 week to 5 months). The cause was infection/suspected infection in 5/8 revisions and they were treated with debridement, antibiotics, irrigation, retention (DAIR), and exchange of the insert. 3 patients required additional surgery, including 1 arthrodesis and 1 amputation. None of the patients died due to their revision surgery (Table 2).

**Table 2.** The 8 revisions and following surgeries after the first revision

	Time after operation	Indication	First revision	Consequent problems following revisions— time after first revision	Months until death
1	1 month	Knee luxation with	TKA	Arthrodesis after 2 weeks	125
		rupture of quadriceps			
2	1 month	Infection	Exchange plastic insert, DAIR	–	46
3	5 months	Patella fracture	Osteosynthesis, exchange	1. Skin necrosis—wound	98
			tibial component and plastic	revision after 2 weeks	
			insert	2. Amputation after 5 weeks	
4	1 month	Patellar tendon rupture	Exchange plastic insert, DAIR	–	(Alive at 54)
		and suspected infection			
5	3 weeks	Infection	Exchange plasticinsert, DAIR	–	(Alive at 58)
6	3 weeks	Infection	Exchange plasticinsert, DAIR	1. Luxation of femorotibial	46
				components—exchange	
				insert after 2 months	
				2. Fracture—osteosynthesis	
				after 2.5 months	
7	1 week	Infection	Exchange plastic insert, DAIR	–	33
8	3 months	Patella luxation	Addition of patellar component	–	46

DAIR: debridement, antibiotics, irrigation and retention.

Both preoperative and 1-year postoperative PRO data were available for 22 of the nonagenarian patients (Figure 1). When PRO scores of the sub-cohort were compared with those of the 65- to 74-year-old primary TKA patients operated on for OA during 2015, the results were similar preoperatively and 1-year postoperatively in all PROs, except for the KOOS symptoms subscale. On the KOOS symptoms subscale, the nonagenarian group reported statistically and clinically significantly fewer symptoms, both preoperatively and 1-year postoperatively, than the younger group (Table 3). The proportion of OMERACT–OARSI responders was 89% in the younger group and three-fourths of the nonagenarians (17/22 patients) were classified as responders. Furthermore, 85% of the younger group and 21/22 of nonagenarian patients were very satisfied or satisfied with the surgery (Table 3).

**Table 3.** Patient-reported outcome preoperatively and 1-year postoperatively in nonagenarians and the SKAR PROM cohort of 65- to 74-year-olds operated with TKA for OA in 2015

	Preoperatively				1-year postoperatively			
	Nonagenarian	65–74 year			Nonagenarian	65–74 year		
	n = 22	n = 1,035	p-value	Difference	n = 22	n = 1,035	p-value	Difference
KOOS–Pain, mean (CI)	47 (38–56)	41 (40–42)	0.2	6 (–3 to 15)	82 (71–92)	82 (81–83)	0.9	–1 (–11 to 10)
KOOS–Symptoms, mean (CI)	61 (51–71)	47 (46–48)	<0.01	14 (4 to 24)	87 (82–93)	79 (78–80)	0.005	9 (3 to 14)
KOOS–ADL, mean (CI)	51 (41–60)	46 (45–47)	0.4	4 (–5 to 14)	76 (67–85)	80 (79–82)	0.3	–5 (–14 to 4)
KOOS-Sports/Rec, mean (CI)	17 (6–27)	13 (12–14)	0.5	4 (–7 to 15)	35 (21–47)	42 (41–44)	0.3	–7 (–22 to 7)
KOOS– QoL, mean (CI)	28 (19–36)	23 (22–24)	0.3	5 (–4 to 13)	75 (64–85)	67 (66–69)	0.2	–8 (–3 to 18)
EQ-VAS, mean (CI)	71 (63–79)	67 (66–69)	0.3	4 (–4 to 12)	76 (68–85)	78 (76–79)	0.7	2 (–10 to 7)
Satisfaction, n (%)					21	878 (84.8)	0.5	
OMERACT-OARSI responder, n (%)					17	925 (89.4)	0.07	

CI =95% confidence interval, KOOS = Knee injury and Osteoarthritis Outcome Score, ADL = activity of daily life function,

Sport/Rec = Sport and recreation function, QoL = quality of life, VAS = visual analogue scale, OMERACT–OARSI = Osteoarthritis Research Society International–Outcome Measures in Rheumatology.

**Figure 4.**
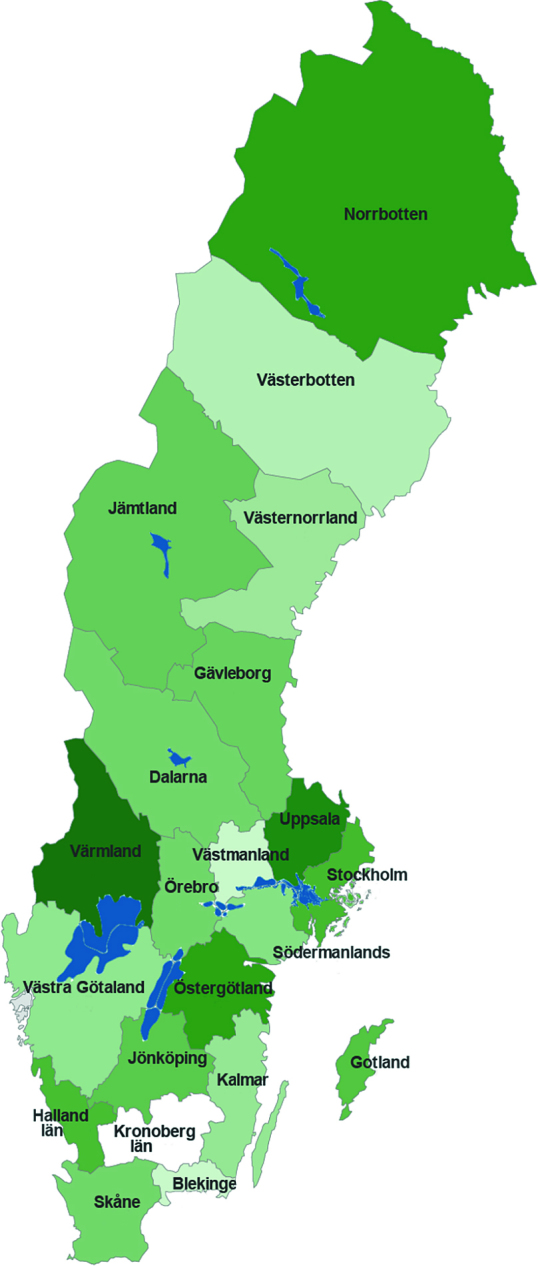
Incidence ratio calculated for each county, using the number of TKAs and inhabitants in each county.

The incidence of primary TKA for OA in nonagenarians was different amongst counties (range, 0–5.1/10,000 inhabitants) (Table 4 and Figure 4).

**Table 4.** Incidence ratio calculated for each county, using the number of TKAs and inhabitants in each county

				Nonagenarian
		Nonagenarian		TKA
	TKAs	TKAs/	Nonagenarian	incidence/
County	2000–1016	year	inhabitants	10,000
Stockholm	89	5.2	15,286	3.4
Uppsala	19	1.1	2,490	4.5
Sörmland	8	0.5	2,404	2.0
Östergötland	26	1.5	3,784	4.0
Jönköping	16	1	3,244	2.9
Kronoberg	0	0	1,888	0
Kalmar	7	0.4	2,502	1.6
Gotland	3	0.2	568	3.1
Blekinge	2	0.1	1,489	0.8
Skåne	45	2.7	11,119	2.4
Halland	15	0.9	2,696	3.3
Västra Götaland	42	2.5	13,617	1.8
Värmland	23	1.4	2,641	5.1
Örebro	11	0.7	2,655	2.4
Västmanland	3	0.2	2,250	0.8
Dalarna	11	0.6	2,698	2.4
Gävleborg	11	0.4	2,524	2.6
Västernorrland	6	0.4	2,157	1.6
Jämtland	6	0.4	1,302	2.7
Västerbotten	4	0.2	1,907	1.2
Norrbotten	12	0.7	1,783	4.0
Total	359	21		

The mean LoS for nonagenarians was somewhat longer (6.2, CI 5.1–7.4) than for the rest of the TKA for OA population (4.1, CI 4.1–4.2) in the region of Skane between January 1, 2007, and December 31, 2016. In addition, there was a noticeable change in the mean LoS for the TKA population during these 10 years (6.2 to 2.8 days).

## Discussion

Improvements in general health, medical services, and social welfare have caused a substantial increase in the nonagenarian population. This will undoubtedly result in a higher number of patients with advanced knee OA. Our results on death rates, revision rates, and PROs suggest that nonagenarians with knee OA who qualify for TKA have good outcomes as compared with the other age groups having TKA. However, the material is small and there may be differences. We found varying incidences (0–5/100,000) of TKA in nonagenarians in different counties in Sweden, which indicates a bias in patient selection.

Our findings concerning death rates, revision rates, and PROs are mostly in line with others (Belmar et al. 1999, Joshi and Gill 2002, Pagano et al. 2004, Karrupiah et al. 2008) (Table 1).

Complications are one of the key factors to be considered when making the decision for TKA in nonagenarian patients. Perioperative complication rates after TKA have been reported as being as high as 11 of 15 TKAs and as low as 5 of 20 TKAs in the nonagenarian population (Belmar et al. 1999, Joshi and Gill 2002). D’Apuzzo et al. (2014) identified more than 58,000 nonagenarians in the USA Nationwide Inpatient Sample (NIS) who had had TKA or THA surgery for various diagnoses and found that age affected the rate of early medical complications after total joint arthroplasty. Although complication rates seem to be higher in nonagenarians than in younger patients undergoing TKA, it is important to point out that almost all of these complications were reported to be typically transient, being resolved in the early postoperative days without long-term sequelae. It should also be kept in mind that the definition of complications, follow-up duration (days versus years), and inclusion of historic data varies substantially amongst studies, which results in various rates of reported complications (Belmar et al. 1999, Joshi and Gill 2002, Pagano et al. 2004, Alfonso et al. 2007, Karrupiah et al. 2008, Petruccelli et al. 2012, Kennedy et al. 2013, D’Apuzzo et al. 2014, Kuperman et al. 2016).

Death rate may be considered as an indicator of safety; however, its use in elective surgery such as TKA in very old patients is limited as these patients naturally have a shorter life expectancy, irrespective of the surgery. Average life expectancy of a 90-year-old Swedish person was 4.2 years in 2016 (Human Mortality Database). In this case, the long-term death rate would be less important compared with patients’ ability to be active and independent in their remaining years. Because of this, early and 1-year postoperative death rates are better indicators for safety. That being said, nonagenarians who are offered or demand a primary TKA are more likely to have better health than their counterparts. As previously shown (Joshi and Gill 2002, Alfonso et al. 2007), it is no surprise that the average life expectancy of nonagenarians who had TKA surpasses the national average of their counterparts. Our cohort with a mean age of 92 years had lower 1-year death rate than the average 92-year-old individual in Sweden in 2016 (7% and 20%) (Human Mortality Database). Another important point to note is that cumulative death rates of the patients in our study at both 5 years and 10 years after the operation were noticeably lower than their 92-year-old counterparts (43% and 73%, 85% and 97% respectively) (Figure 3). This shows that more than half of the nonagenarian patients can expect to live more than 5 years with their implant. Our findings on death rates were in line with the literature (Belmar et al. 1999, Joshi and Gill 2002, Pagano et al. 2004, Karrupiah et al. 2008) (Table 1). Similar death rates were found in reports studying both TKA and THA in nonagenarians (Alfonso et al. 2007, Petruccelli et al. 2012). Petruccelli et al. (2012) showed in a literature review consisting of 105 total arthroplasties in nonagenarians that the perioperative death rate was 2%, and 1-year postoperative death rate was 8%, while D’Apuzzo et al. (2014) showed that the US cohort of 58,000 nonagenarians operated on with total arthroplasty had a substantially higher in-hospital death rate (3%) than younger patients (45–89 years, 0.2%) even after adjusting for comorbidities. The higher rate may be the result of their material including several indications for arthroplasty such as hip fractures and rheumatoid arthritis.

Revision rates for primary TKA in the nonagenarians have not been thoroughly analyzed in the literature, probably due to a limited number of patients included (Belmar et al. 1999, Joshi and Gill 2002, Pagano et al. 2004, Karrupiah et al. 2008) (Table 1). In our study, 8 of 329 (2.4%) patients had revision surgery, all within the first 5 months after the primary TKA. Their first revisions probably did not affect these patients’ death rate, as they lived for another 2–10 years after the first revision.

Our study showed a comparable outcome regarding pain to the other nonagenarian studies reporting patient/surgeon-reported outcomes, while those studies reported less improvement in function (Belmar et al. 1999, Joshi and Gill 2002, Pagano et al. 2004, Karrupiah et al. 2008) (see Table 1). Alfonso et al. (2007) suggested the reason for lower functional improvement might be other conditions such as systemic disease, poor balance, and impaired vision rather than the inefficiency of TKA. However, these studies, except for the study by Karrupiah et al. (2008), probably reported the surgeon’s opinions with the earlier KSS rather than the patients’ experiences. It is also important to point out that the nonagenarians in our study reported fewer symptoms in the KOOS both preoperatively and 1-year postoperatively than the younger age group; it could be speculated that nonagenarians may have had more disabling/pronounced symptoms from other parts of the body than those from the knee or, owing to the bias in patient selection, older patients may have had a more positive attitude in life and thus experienced less pain and fewer symptoms than the average patient (Thompson et al. 2018). Satisfaction with the surgery was high (21 of 22) and most of the patients were classified as OMERACT–OARSI responders (17 of 22). However, TKA surgery hardly improved the EQ-VAS in both nonagenarian and 65- to 74-year-old group, preoperatively to 1-year postoperatively according to our definition of clinical relevance. The reason may be that TKA surgery is not expected to improve the health in this group of patients.

The variation in the regional incidence of TKA in Sweden indicates possible discrimination against nonagenarians amongst orthopedic surgeons in different counties. In addition, if general practitioners lack belief in knee arthroplasty in nonagenarians these patients are not referred to the orthopedic surgeon, which could contribute to the patient selection bias. This has rarely been discussed in the literature. In a prospective cohort study by Hamel et al. (2008), 174 patients with severe radiographic knee and hip OA were identified. During 1-year follow-up they reported 123 of the patients did not undergo TKA. Half of these patients reported that surgery had never been suggested, and the rest had been hesitant to have surgical treatment. Patients who were not operated were reported to be older than the rest of the cohort, indicating age discrimination. Our data on this topic may help both the surgeon and the patient to make an informed decision.

The burden on health resources can also be debated while deciding on elective surgery in the elderly population. Fewer years of expected life may seem like a limiting factor for the cost-effectiveness of the surgery. Our data from the last 10 years showed that nonagenarian patients’ LoS was 2 days longer compared with those who were younger, which is associated with higher costs per TKA. In addition, there was a noticeable change in the mean LoS for the TKA cohort in this study during these 10 years (6.2 to 2.8 days), which reflects the change in healthcare trends. In Sweden, the average cost of TKA for 90- to 94-year-old patients was US$7,810 in 2016 (Swedish Association of Local Authorities and Regions 2017). This amount should be compared with the possible economic burden caused by keeping a healthy, active patient away from TKA. Pain, loss of strength, and lower bone density are few of the problems that can diminish the ability of nonagenarians to live independently. Karrupiah et al. (2008) have assumed that nonagenarian patients with severe OA would have required nursing home admission without the surgery, as 15% of all patient admissions to nursing homes are due to immobility secondary to arthritis (Guccione et al. 1989). In countries where the government finances social care; delaying nursing home placement may reduce the burden on health resources (Karrupiah et al. 2008). This might be suggested to be the case for Sweden because the average annual cost per person in a nursing home is US$90,561 (Swedish Association of Local Authorities and Regions 2018).

We acknowledge limitations of this study. Clinical information (i.e., comorbidities, amount of blood loss, medical complications) was not available in the SKAR, which precluded analysis of risks associated with death. Another limitation is that PRO data have been collected since 2009 in the SKAR and the data collection is not compulsory, which reduced the number of patients for PRO analysis.

In summary, our study emphasizes that TKA is a safe procedure with good outcome in nonagenarians with OA. Our findings may help surgeons and patients assessing the risks and outcome associated with the procedure.

The study was conceived by LL. OR, AWD, and EAS performed the analyses and LL and EAS wrote the initial draft. All the authors contributed to the interpretation of the data and to revision of the manuscript.

The authors would like to thank Sofia Löfvendal for contribution to the discussion on health economics.

*Acta* thanks Ove Furnes and Sten Rasmussen for help with peer review of this study.

## References

[CIT0001] Alfonso D T, Howell R D, Strauss E J, et al. Total hip and knee arthroplasty in nonagenarians. J Arthroplasty 2007; 22 (6): 807. 17826269 10.1016/j.arth.2006.10.016

[CIT0002] Belmar C J, Barth P, Lonner J H, et al. Total knee arthroplasty in patients 90 years of age and older. J Arthroplasty 1999; 14 (8): 911. 10614879 10.1016/s0883-5403(99)90002-5

[CIT0003] Biau D, Mullins M M, Judet T, Piriou P Is anyone too old for a total knee replacement. Clin Orthop Relat Res 2006; 448(448): 180–4. 16826114 10.1097/01.blo.0000194682.33000.f9

[CIT0004] Culliford D, Maskell J, Judge A, Cooper C, Prieto-Alhambra D, Arden N K Future projections of total hip and knee arthroplasty in the UK: results from the UK clinical practice research datalink. Osteoarthritis Cartilage 2015; 23: 594–600. 25579802 10.1016/j.joca.2014.12.022

[CIT0005] D’Apuzzo M R, Pao A W, Novicoff W M, Browne J A Age as an independent risk factor for postoperative morbidity and mortality after total joint arthroplasty in patients 90 years of age or older. J Arthroplasty 2014; 29 (3): 477–80. 24029720 10.1016/j.arth.2013.07.045

[CIT0006] Easterlin M C, Chang D G, Talamini M, Chang D C Older age increases short-term surgical complications after primary knee arthroplasty. Clin Orthop Relat Res 2013; 471 (8): 2611–20. 23613088 10.1007/s11999-013-2985-8 PMC3705042

[CIT0007] EuroQol Group EQ-5D EuroQol Group website. http://www.euroqol.org; 2015.

[CIT0008] Guccione A A, Meenan R F, Anderson J J Arthritis in nursing home residents: a validation of its prevalence and examination of its impact on institutionalization and functional status. Arthritis Rheum 1989; 32: 1546–53. 2597209 10.1002/anr.1780321208

[CIT0009] Hamel M B, Toth M, Legedza A, Rosen M P Joint replacement surgery in elderly patients with severe osteoarthritis of the hip or knee: decision making, postoperative recovery, and clinical outcomes. Arch Intern Med 2008; 168 (13): 1430–40. 18625924 10.1001/archinte.168.13.1430

[CIT0010] Human Mortality Database University of California, Berkeley (USA), and Max Planck Institute for Demographic Research (Germany). Available at www.mortality.org or www.humanmortality.de (data downloaded on 01.04.2018).

[CIT0011] Inacio M C S, Paxton E W, Graves S E, Namba R S, Nemes S Projected increase in total knee arthroplasty in the United States: an alternative projection model. Osteoarthritis Cartilage 2017; 25 (11): 1797–1803. 28801208 10.1016/j.joca.2017.07.022

[CIT0012] Joshi A B, Gill G Total knee arthroplasty in nonagenarians. J Arthroplasty 2002; 17 (6): 681. 12216019 10.1054/arth.2002.32175

[CIT0013] Karrupiah S V, Banaszkiewicz P A, Ledingham W M The mortality, morbidity and cost benefits of elective total knee arthroplasty in the nonagenarian population. Int Orthop 2008; 32 (3): 339–43. 17333185 10.1007/s00264-007-0324-y PMC2323413

[CIT0014] Kennedy J W, Johnston L, Cochrane L, Boscainos P J Total knee arthroplasty in the elderly: does age affect pain, function or complications? Clin Orthop Relat Res 2013; 471: 1964–9. 23354464 10.1007/s11999-013-2803-3 PMC3706666

[CIT0015] Koh I J, Kim T K, Chang C B, Cho H J, In Y Trends in use of total knee arthroplasty in Korea from 2001 to 2010. Clin Orthop Rel Res 2013; 471 (5): 1441–50. 10.1007/s11999-012-2622-y PMC3613551 23054516

[CIT0016] Kuperman E F, Schweizer M, Joy P, Gu X, Fang M M The effects of advanced age on primary total knee arthroplasty: a meta-analysis and systematic review. BMC Geriatrics 2016; 16: 41. 26864215 10.1186/s12877-016-0215-4 PMC4750247

[CIT0017] Kurtz S M, Ong K L, Lau E, Bozic K J. Impact of the economic downturn on total joint replacement demand in the United States. J Bone Joint Surg (Am) 2014; 96-A: 624–30. 10.2106/JBJS.M.00285 24740658

[CIT0018] Laskin R S Total knee replacement in patients older than 85 years. Clin Orthop Relat Res 1999; 367: 43–9. 10546597

[CIT0019] Nemes S, Roflson O, W-Dahl A, Garellick G, Sundberg M, Karrholm J, Robertsson O Historical view and future demand for knee arthroplasty in Sweden. Acta Orthop 2015; 86 (4): 426–31. 25806653 10.3109/17453674.2015.1034608 PMC4513596

[CIT0020] Niemelainen M J, Makela K T, Robertsson O, W-Dahl A, Furnes O, Fenstad A M, Pedersen A B, Schroder H M, Huhtala H, Eskelinen A Different incidences of knee arthroplasty in the Nordic countries. Acta Orthop 2017; 88 (2): 173–8. 28056570 10.1080/17453674.2016.1275200 PMC5385112

[CIT0021] Pagano M W, McLamb L A, Trousdale R T Total knee arthroplasty for patients 90 years of age and older. Clin Orthop Relat Res 2004; (418): 179. 10.1097/00003086-200401000-00029 15043112

[CIT0022] Petruccelli D, Rahman W A, de Beer J, Winemaker M Clinical outcomes of primary total joint arthroplasty among nonagenarian patients. J Arthroplasty 2012; 27 (9): 1599–1603. 10.1016/j.arth.2012.03.007 22552218

[CIT0023] Pham T, van der Heijde D, Altman R D, Anderson J J, Bellamy N, Hochberg M, Simon L, Strand V, Woodworth T, Dougados M OMERACT–OARSI initiative: osteoarthritis Research Society International set of responder criteria for osteoarthritis clinical trials revisited. Osteoarthritis Cartilage 2004; 12 (5): 389–99. 10.1016/j.joca.2004.02.001 15094138

[CIT0024] Roos E M, Roos H P, Lohmander L S, Ekdahl C, Beynnon B D Knee Injury and Osteoarthritis Outcome Score (KOOS): development of a self-administered outcome measure. J Orthop Sports Phys Ther 1998; 28(2): 88–96. 9699158 10.2519/jospt.1998.28.2.88

[CIT0025] SKAR Swedish Knee Arthroplasty Register Annual Report 2017. 2017; ISBN 978-91-8807-15-4.

[CIT0026] Statistics Sweden The future population of Sweden 2018–2070. Demographic reports 2018:1.

[CIT0027] Swedish Association of Local Authorities and Regions. The Swedish Cases Costing Database (KPP) 2017. skl.se/ekonomijuridikstatistik/statistik/kostnadperpatientkpp/kppdatabas.1079 (data downloaded on 25 05 2018).

[CIT0028] Swedish Association of Local Authorities and Regions. Öppna jämförelser 2017 Vård och omsorg om äldre: jämförelser mellan kommuner och län. 2018; ISBN 978-91-7875-674-2.

[CIT0029] Thompson K A, Bulls H W, Sibille K T, Bartley E J, Glover T L, Terry E L, Vaughn I A, Cardoso J S, Sotolongo A, Staud R, Hughes L B, Edberg J C, Redden D T, Bradley L A, Goodin B R, Fillingim R B Optimism and psychological resilience are beneficially associated with measures of clinical and experimental pain in adults with or at risk for knee osteoarthritis. Clin J Pain 2018; [published online ahead of print Jul 21]. doi: 10.1097/AJP.0000000000000642. PMC6219932 30036216

[CIT0030] Turkiewicz A, Gerhardsson de Verdier M, Engström G, Nilsson P M, Mellström C, Lohmander S, Englund M Prevalence of knee pain and knee OA in southern Sweden and the proportion that seeks medical care. Rheumatology 2014; 54: 827–35. 25313145 10.1093/rheumatology/keu409

[CIT0031] Williams D P, Price A J, Beard D J, Hadfield S G, Arden N K, Murray D W, Field R E The effects on patient-reported outcome measures in total knee replacements. Bone Joint J 2013; 95-B: 38–44. 23307671 10.1302/0301-620X.95B1.28061

